# Fusarubin and Anhydrofusarubin Isolated from A *Cladosporium* Species Inhibit Cell Growth in Human Cancer Cell Lines

**DOI:** 10.3390/toxins11090503

**Published:** 2019-08-29

**Authors:** Sabrina Adorisio, Alessandra Fierabracci, Isabella Muscari, Anna Marina Liberati, Lorenza Cannarile, Trinh Thi Thuy, Tran Van Sung, Hossain Sohrab, Choudhury Mahmood Hasan, Emira Ayroldi, Carlo Riccardi, Abdul Mazid, Domenico V. Delfino

**Affiliations:** 1 Department of Medicine, Foligno Nursing School, Via Oberdan 123, 06034 Foligno (PG), Italy; 2 Infectivology and Clinical trials Area, Children’s Hospital Bambino Gesù, Via S. Paolo 15, 00146 Rome, Italy; 3 Department of Medicine, Section of Onco-hematology, S. Maria Terni Hospital, Via Tristano Di Joannuccio, 05100 Terni, Italy; 4 Section of Pharmacology, Department of Medicine, University of Perugia, Piazzale Severi, S.Andrea delle Fratte, 06129 Perugia, Italy; 5 Institute of Chemistry, Vietnam Academy of Science and Technology, Hanoi 10000, Vietnam; 6 BCSIR Laboratories Dhaka, Dr. Qudrat-I-Khuda Road, Dhanmondi, Dhaka 1205, Bangladesh; 7 Department of Pharmaceutical Chemistry, Faculty of Pharmacy, University of Dhaka, Dhaka 1000, Bangladesh

**Keywords:** *Cladosporium*, *Rauwolfia serpentina*, fusarubin, anhydrofusarubin, endophytic fungi, p21, caspase-8, human cancer cell lines

## Abstract

*Cladosporium* species are endophytic fungi that grow on organic matter and are considered food contaminants. The anti-microbial and anti-tumor naphthoquinones fusarubin (FUS) and anhydrofusarubin (AFU) were isolated using column chromatography from a *Cladosporium* species residing inside *Rauwolfia* leaves. The impact of FUS and AFU on cell growth was assessed in acute myeloid leukemia (OCI-AML3) and other hematologic tumor cell lines (HL-60, U937, and Jurkat). Treatment with FUS or AFU reduced the number of OCI-AML3 cells as evaluated by a hemocytometer. Flow cytometry analyses showed that this effect was accompanied by diverse impairments in cell cycle progression. Specifically, FUS (20 or 10 μg/mL significantly decreased the percentage of cells in S phase and increased the percentage of cells in G2/M phase, whereas AFU increased the percentage of cells in G0/G1 phase (50 and 25 μg/mL) and decreased the percentage of cells in S (50 μg/mL) and G2/M (50 and 25 μg/mL) phases. Both substances significantly increased apoptosis at higher concentrations. The effects of FUS were more potent than those of AFU, with FUS up-regulating p21 expression in a p53-dependent manner, as detected by Western blot analyses, likely the consequence of decreased ERK phosphorylation and increased p38 expression (both of which increase p21 stability). FUS also decreased Akt phosphorylation and resulted in increased Fas ligand production and caspase-8/3-dependent apoptosis. These results suggest that FUS and AFU inhibit proliferation and increase apoptosis in cell lines derived from hematological cancers.

## 1. Introduction

Endophytic fungi interact with host plants and alter aspects of their physiology [1], conferring increased tolerance to stress [2], improving immune system function [3], bolstering defenses against disease [4], and aiding substance absorption [5]. Thus, the possibility of exploiting endophytic fungi as biocontrol agents has received increasing attention [6,7,8]. Endophytic *Cladosporium* species, such as *Cladosporium fulvum*, allow the expression of plant genes that code for resistance to pathogens, providing long-term protection [9]. These endophytic fungi cause human diseases such as unilateral cervical lymphadenopathy [10] and acne-like subcutaneous phaeohyphomycosis [11]. However, they can also be used to derive bioactive compounds, such as the anti-cancer drug paclitaxel [12]. Thus, the toxicity of fungi and their propensity to cause disease is particularly dangerous because they can be contaminants of different types of food [13]. Thus, studying the interaction between fungi such as *Cladosporium* sp. and mammalian cells is important: (1) to avoid the putative toxicity of their metabolites; and (2) to identify novel bioactive compounds and dissect their mechanisms with the aim of developing new therapeutic approaches to cancer. Recently, two naphthoquinones, anhydrofusarubin (AFU) and the methyl ether fusarubin (FUS) were isolated from a *Cladosporium* species growing on the leaves of *Rauwolfia serpentina* (L.) Benth. Ex Kurz. These two compounds show potential cytotoxicity against human leukemia cells, with FUS exhibiting activity against *Staphylococcus aureus*, *Escherichia coli*, *Pseudomonas aeruginosa*, and *Bacillus megaterium*, suggesting that these compounds could be useful for developing new anti-microbial and anti-cancer drugs [12]. Although promising, these results are preliminary and do not reveal a mechanism of action that could be exploited in anti-cancer therapy or that might induce toxic effects against healthy cells. 

Two important anti-tumor and toxic mechanisms are the ability to undergo continuous proliferation and to evade apoptosis [14]. One of the more common anti-proliferative mechanisms is based on p21, which is involved in DNA repair and other functions critical for normal cell growth. For example, p21 inhibits cyclin-dependent kinase function, thereby blocking the cell cycle, and is also involved in transcription, cell death, and motility [15]. Expression of p21 protects cells from toxic stimuli induced by different chemicals [15]. p21 function is critical for the life of cells, particularly when considered together with p53 function [16]. In the presence of normal or augmented p53 protein, p21 determines whether a cell enters either a transient cell cycle arrest and growth inhibition with consequent error-free DNA repair and normal replication or a chronic state of senescence or apoptosis. Both mechanisms protect cells from cancer and result in a low level of genomic instability [16]. In light of the importance of the p21/p53 system, the identification of novel compounds that can interfere with this system is critical for therapeutically enhancing cell protection. 

In this work, we analyzed in detail how FUS and AFU affect proliferation and apoptosis to better understand their biological actions. We found that both substances interfered with the cell cycle and increased apoptosis in hematological cancer cell lines. In particular, FUS exerted its effect by increasing expression of p21 and p53 and activating the caspase-8/3 apoptotic pathway [17]. Therefore, these novel compounds are potentially useful for modulating vital cell activities.

## 2. Results

### 2.1. Dose-Dependent Effects of FUS and AFU on OCI-AML3 Cell Number

**Figure 1:**
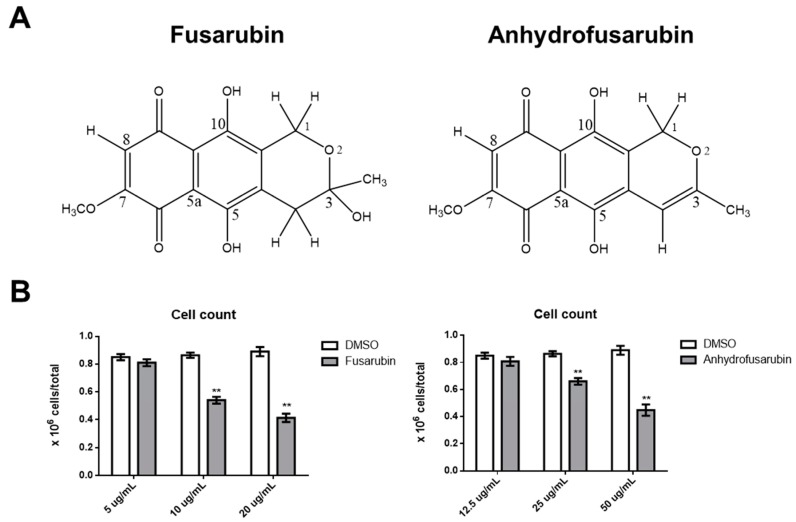
Chemical structures and effects of FUS and AFU on OCI-AML3 cell number. (**A**) Chemical structure of FUS and AFU. (**B**) Bars represent the number of viable cells counted after 24 h of treatment with vehicle (DMSO), FUS (left panel), or AFU (right panel) at the concentrations reported on the *x*-axis. Data from five independent experiments are reported as mean ± standard error of the mean (SEM). ** *p* < 0.01.

We first tested whether FUS and AFU affected the growth of acute myeloid leukemia OCI (OCI-AML3) cells. Figure 1 shows that after 24 h of treatment with 10 or 20 μg/mL FUS or with 25 or 50 μg/mL AFU, the number of OCI-AML3 cells was significantly lower than that after treatment with control vehicle, dimethyl sulfoxide (DMSO). The half maximal inhibitory concentration (IC_50_) of FUS and AFU was 16.1 and 45.5 μg/mL, respectively. 

### 2.2. Effects of FUS and AFU on Cell Death and Cell Cycle Progression

**Figure 2:**
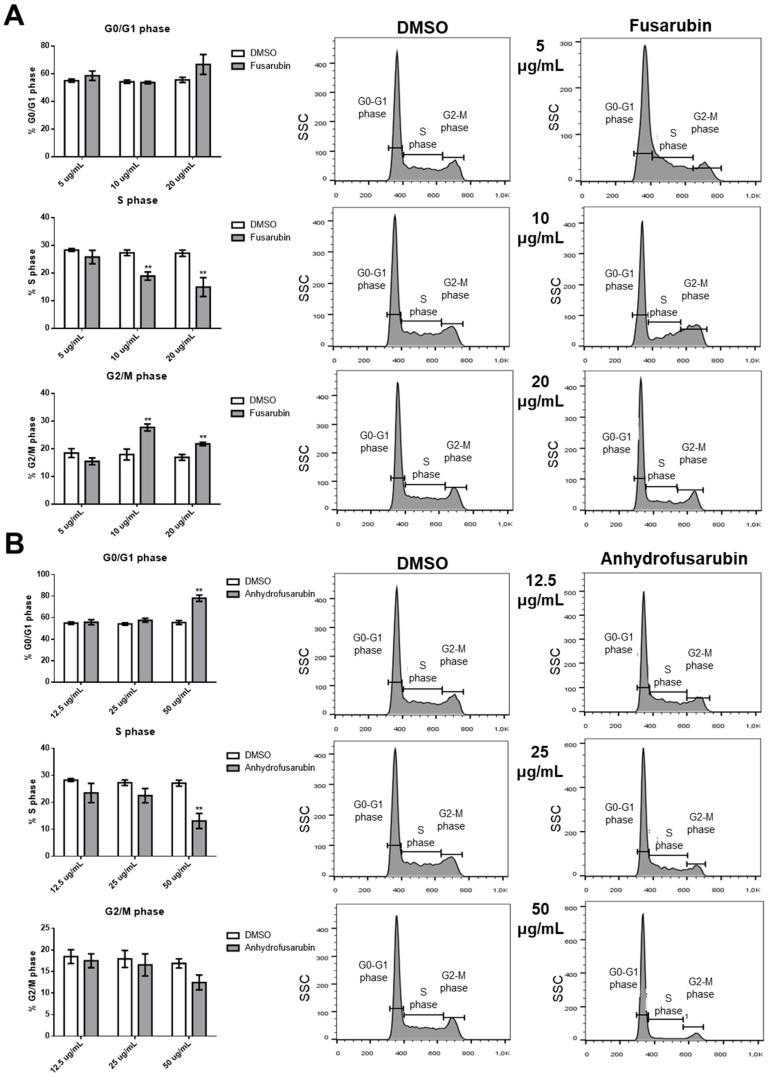
Effects of FUS and AFU on OCI-AML3 cell cycle progression. Bars represent the percentage of cells in G0/G1, S, or G2/M phase after 24 h of treatment with control vehicle (DMSO), (**A**) FUS, or (**B**) AFU at the concentrations reported on the *x*-axis. Right panels show flow cytometry analyses of representative experiments. Data from three independent experiments are reported as mean ± SEM. ** *p* < 0.01.

**Figure 3:**
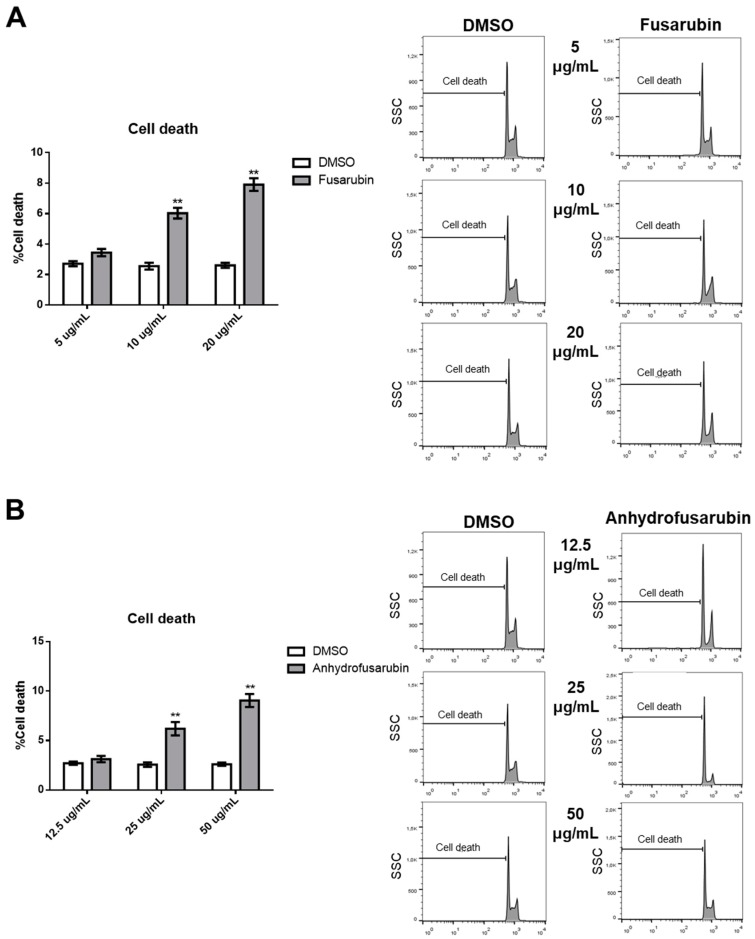
Effects of FUS and AFU on OCI-AML3 cell death. Bars represent percentage of cell death after 24 h of treatment with control vehicle (DMSO), (**A**) FUS, or (**B**) AFU at the concentrations reported on the *x*-axis. Right panels show flow cytometry analyses of representative experiments. Data from three independent experiments are reported as mean ± SEM. ** *p* < 0.01.

The FUS- and AFU-induced reductions in cell number may be due to increased cell death, decreased proliferation, or both. To evaluate these processes, we stained cell nuclei with propidium iodide (PI) and performed flow cytometry analysis to investigate cell cycle status and the death of FUS- or AFU-treated cells. As shown in Figure 2A, FUS treatment (20 and 10 μg/mL) significantly decreased the percentage of cells in S phase and increased the percentage of cells in G2/M phase. By contrast, as shown in Figure 2B, AFU treatment (50 μg/mL) significantly increased the percentage of cells in G0/G1 phase and decreased the percentage of cells in S phase. Figure 3 shows that when cell death was analyzed under the same conditions, FUS (10 and 20 μg/mL) and AFU (25 and 50 μg/mL) significantly increased cell death. These results suggest that AFU and FUS reduce OCI-AML3 cell number by inducing cell cycle arrest and increasing cell death, although the effects of FUS are more potent.

### 2.3. Effects of FUS and AFU on Cell Proliferation Pathways

**Figure 4:**
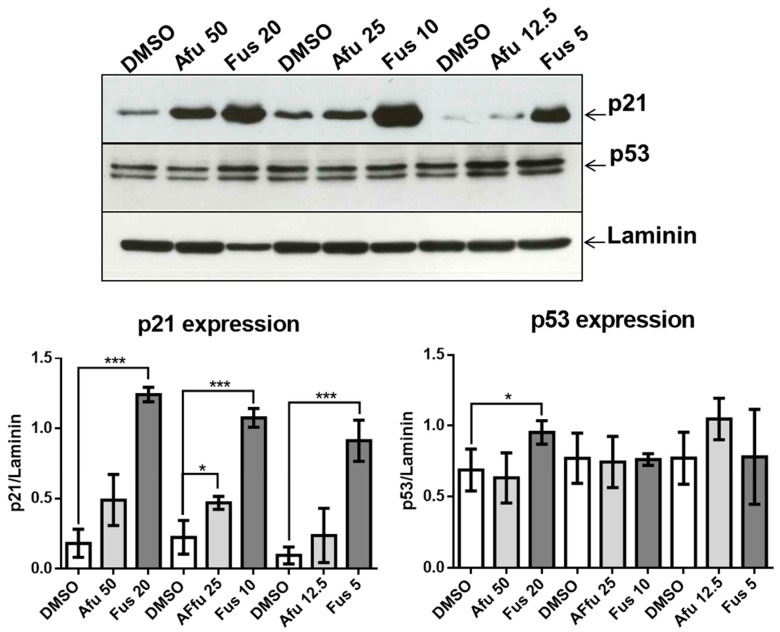
Effects of FUS and AFU on expression of proteins involved in the cell cycle. (Upper panels) Western blot analysis illustrating expression of p21, p53, and laminin using cell lysates extracted from OCI-AML3 cells treated with vehicle (DMSO), FUS, or AFU for 24 h. Numbers represent concentrations in μg/mL. Western blots are representative of three independent experiments. (Lower panels) Quantification of experiments shown in upper panels. Data from three independent experiments are reported as mean ± SEM. * *p* < 0.05, *** *p* < 0.001.

We next analyzed potential mechanisms by which FUS and AFU affected the cell cycle using Western blotting to measure expression of p21 in FUS- or AFU-treated OCI-AML3 cells. As shown in Figure 4, FUS significantly upregulated p21 at all three tested concentrations, whereas AFU significantly upregulated p21 only at a concentration of 25 μg/mL. Because p21 is regulated by p53 [18,19], we also investigated whether FUS or AFU treatment induces p53 expression in OCI-AML3 cells. We found that FUS significantly increased expression of p53 only at a concentration of 20 μg/mL, suggesting that p53-dependent pathways are partially responsible for the effect of high FUS concentrations on cell cycle arrest.

### 2.4. Effects of FUS and AFU on Regulation of p21 Function

**Figure 5:**
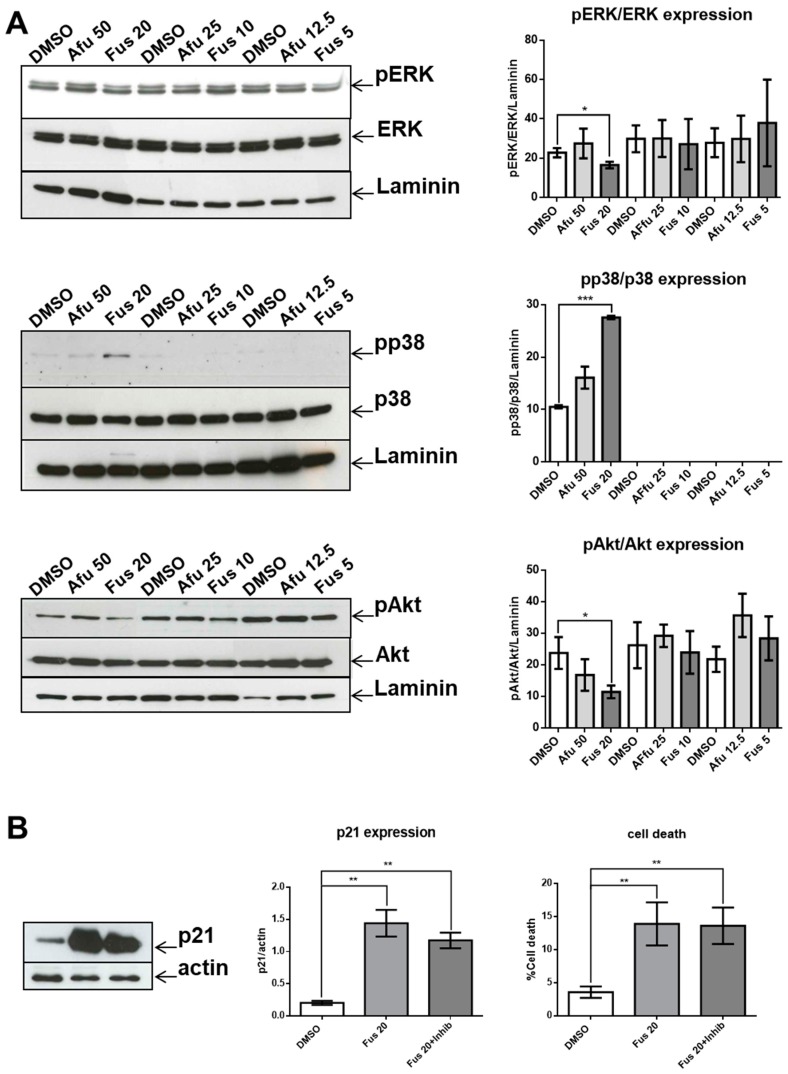
Effects of FUS and AFU on the MAPK pathway. (**A**) Western blot analysis of phosphorylated ERK (pERK), total ERK (ERK), phosphorylated p38 (pp38), total p38 (p38), phosphorylated Akt (pAkt), and total Akt (Akt) expression in OCI-AML3 cells treated with vehicle (DMSO), FUS, or AFU for 24 h. Laminin served as a loading control. Western blots are representative of three independent experiments. (**B**) Western blot analysis of p21 expression in OCI-AML3 cells treated with vehicle (DMSO), FUS at 20 μg/mL (Fus 20), or FUS at 20 μg/mL with p38 inhibitor (Fus 20 + inhib) for 24 h. Actin served as a loading control. Western blots are representative of five independent experiments. Bar graphs show the quantification of experiments shown in (**A**,**B**). Numbers represent concentrations in μg/mL. Data from three (**A**) or five (**B**) independent experiments are reported as mean ± SEM. * *p* < 0.05, ** *p* < 0.01, *** *p* < 0.001.

We investigated the potential roles of ERK, p38, and Akt in FUS- and AFU-induced p21 expression using Western blotting. Figure 5 shows that levels of phosphorylated ERK and Akt significantly decreased after FUS treatment (20 μg/mL) compared with DMSO treatment. Conversely, levels of phosphorylated p38 significantly increased after FUS treatment. AFU did not affect ERK, Akt, or p38 phosphorylation at any tested concentration. Thus, FUS-dependent changes in p21 expression in OCI-AML3 cells are likely related to changes in MAPK pathway signaling, at least at a concentration of 20 μg/mL. To determine whether p21 expression and cytotoxicity are dependent on p38 MAPK activation, we performed additional experiments in which p38 inhibitor was added to the cultures together with FUS. As shown in Figure 5, inhibition of p38 did not significantly decrease p21 expression or apoptosis. 

### 2.5. Effects of FUS and AFU on Apoptotic Pathways

**Figure 6:**
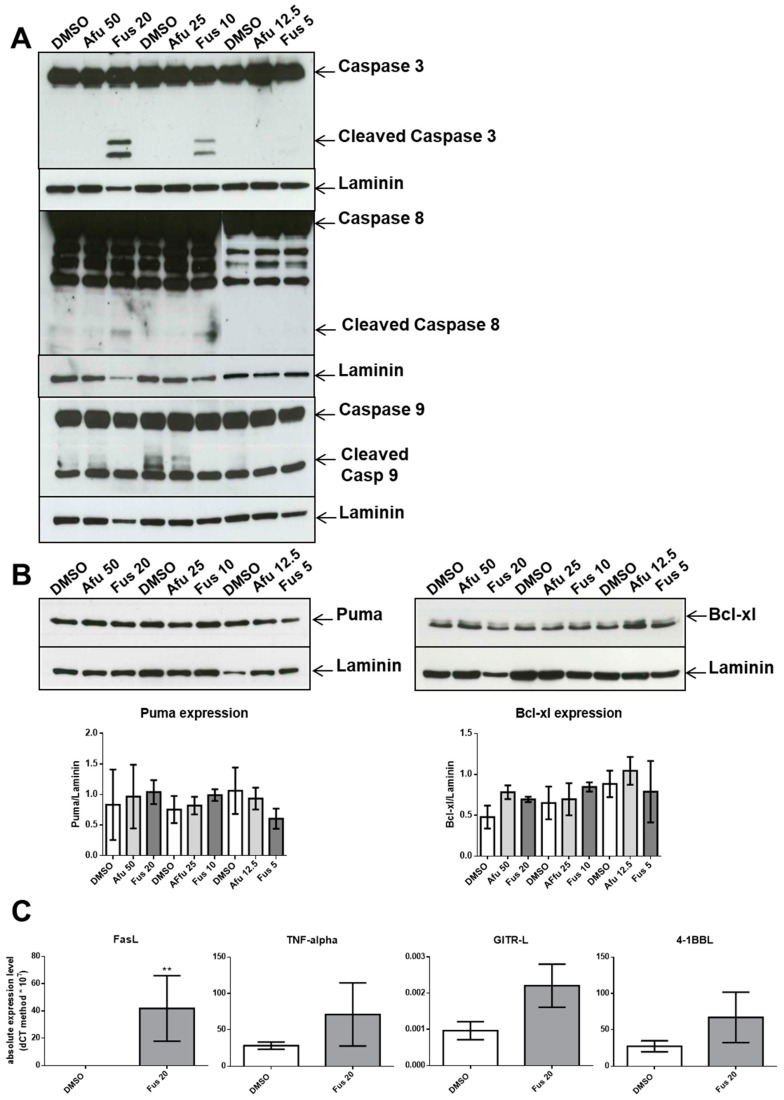
Effects of FUS and AFU on apoptotic pathways. (**A**) Western blot analysis of pro-caspase-3 (caspase-3), activated caspase-3 (cleaved caspase 3), pro-caspase-8 (caspase-8), activated caspase-8 (cleaved caspase 8), pro-caspase-9 (caspase 9), and activated caspase-9 (cleaved caspase 9) in OCI-AML3 cells treated with vehicle (DMSO), FUS, or AFU at different concentrations for 24 h. Western blots are representative of three independent experiments. (**B**) Western blot analysis of PUMA or Bcl-xL and laminin. Quantification of band intensity is shown below each blot. Numbers represent concentrations in μg/mL. Western blots are representative of three independent experiments; bar graphs (mean ± SEM) show the quantification of bands compared with the housekeeping gene, laminin. (**C**) Real-time PCR analyses from five independent experiments (mean ± SEM) of FasL, TNF-alpha, GITR ligand (GITR-L), and 41BB ligand (41BB-L) in cells treated with vehicle (DMSO) or 20 μg/mL FUS. ** *p* < 0.01.

The increased FUS- and AFU-mediated death of OCI-AML3 cells prompted us to investigate the role of the caspase cascade, which is involved in apoptotic cell death. We first examined caspase-3, a terminal caspase involved in apoptosis. We cultured OCI-AML3 cells for 24 h before extracting proteins for Western blotting. Figure 6A shows that activated cleaved caspase-3 was present after treatment with 10 or 20 μg/mL FUS. By contrast, AFU did not induce caspase-3 activation at any tested concentration. These results indicate that FUS-induced death of OCI-AML3 cells is associated with augmented caspase-3 activation. 

The executioner caspase-3 is activated by at least two different pathways. The mitochondrial (i.e., intrinsic) pathway leads to sequential release of cytochrome c from mitochondria and activation of caspase-9, which directly cleaves and activates caspase-3. The second (i.e., extrinsic) pathway involves activation of caspase-8, which also directly cleaves and activates caspase-3 [20]. We analyzed both pathways to determine the mechanism of caspase-3 activation in FUS- and AFU-treated OCI-AML3 cells. Figure 6A shows that caspase-8 was activated following treatment with 10 or 20 μg/mL FUS but was not activated by AFU at any tested concentration; caspase-9 was not activated by FUS or AFU. Interestingly, both FUS and AFU inhibited caspase-9 activation in DMSO-treated cells. Therefore, FUS-induced, but not AFU-induced, cell death is likely dependent on activation of the caspase-8 apoptotic pathway. Because activation of caspase-9 was inhibited by both FUS and AFU, we tested whether FUS- or AFU-induced cell death involved the intrinsic pathway. by measuring the expression of two additional intrinsic apoptotic pathway molecules, PUMA and Bcl-xL, using Western blot analysis. Figure 6B shows that neither FUS nor AFU treatment affected their expression, suggesting that FUS- and AFU-dependent cell death does not occur via the intrinsic pathway. Additionally, as FUS activated caspase-8, we analyzed four members of the TNF superfamily to determine whether they are involved in the caspase-8-dependent extrinsic apoptotic pathway. As shown in Figure 6C, FUS significantly increased the production of Fas ligand (FasL) but not TNF, GITR, or 41BB ligands. Thus, FasL may be responsible for FUS-dependent caspase-8 activation and increased apoptosis.

### 2.6. Effects of FUS on Additional Hematologic Tumor Cell Lines

**Figure 7:**
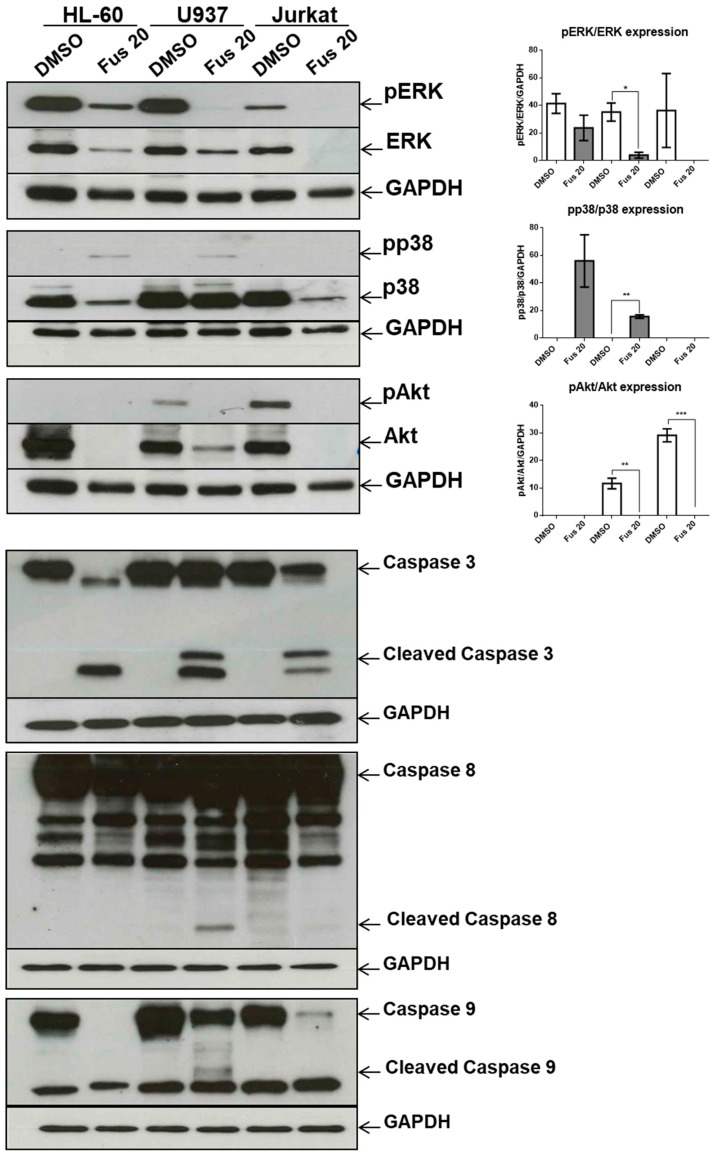
Effects of FUS on different cancer cell lines. Western blot analysis of phosphorylated ERK (pERK), total ERK (ERK), phosphorylated p38 (pp38), total p38 (p38), phosphorylated Akt (pAkt), and total Akt (Akt). GAPDH served as a loading control. Bar graphs show quantification of the three phosphorylated proteins. Western blot analysis of pro-caspase-3 (caspase 3), activated caspase-3 (cleaved caspase 3), pro-caspase-8 (caspase 8), activated caspase-8 (cleaved caspase 8), pro-caspase-9 (caspase 9), and activated caspase-9 (cleaved caspase 9). GAPDH served as a loading control, and DMSO was used as control vehicle. Numbers represent concentrations in μg/mL. Data from three independent experiments are reported as mean ± SEM. * *p* < 0.05, ** *p* < 0.01, *** *p* < 0.001.

Because FUS had a more potent effect on cell death than AFU, we tested its effects on additional hematologic cell lines, including HL-60 and U937 acute myeloid leukemia cell lines and Jurkat cells, a T-cell lymphoma-derived cell line. Figure 7 shows that treatment with 20 μg/mL FUS significantly decreased phosphorylation of ERK and increased phosphorylation of p38 in U937 cells and also decreased Akt phosphorylation in both U937 and Jurkat cells. However, HL60 cells were not affected by FUS treatment. Figure 7 shows that caspase-3 was cleaved and activated in all three FUS-treated cell lines, whereas caspase-8 and caspase-9 were only activated by FUS in U937 cells. Notably, FUS treatment decreased expression of pro-caspase-9 in HL-60, U937, and Jurkat cells but not in OCI-AML3 cells. Together, these results suggest that FUS induces differential regulation of MAPK and apoptotic signaling in different cell lines.

### 2.7. Effects of FUS on Primary Hematologic Cells

**Figure 8:**
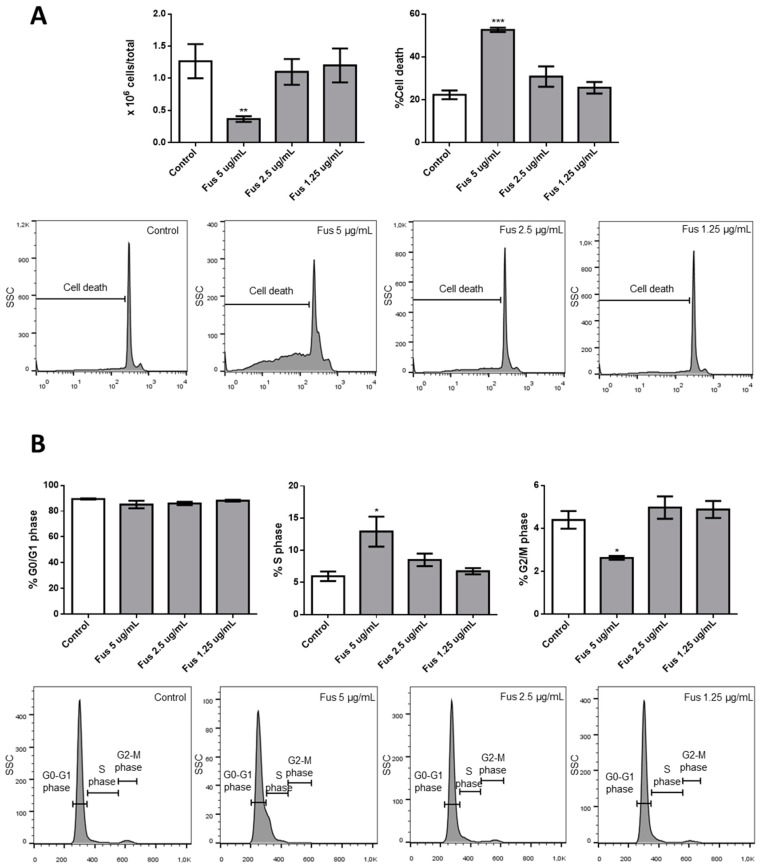
Effects of FUS on primary bone marrow cells. (**A**) Bar graphs show the number of cells or percentage of cell death. Bone marrow cells were cultured with or without different concentrations of FUS. Lower panels show a representative flow cytometry experiment evaluating bone marrow cell death after culturing with or without different concentrations of FUS. (**B**) Percentage of cells in G0/G1, S, or G2/M phase after 24 h of treatment with control vehicle or FUS at concentrations reported on the *x*-axis. Lower panels show a representative experiment evaluating cell cycle progression. Data from three independent experiments are reported as mean ± SEM. * *p* < 0.05, ** *p* < 0.01, *** *p* < 0.001.

Finally, we tested the effects of FUS on primary hematological cells by culturing mouse bone marrow cells with or without FUS. As shown in Figure 8, bone marrow cells were more sensitive than OCI-AML3 cells. Specifically, 5 μg/mL FUS reduced the number of cells, increased apoptosis, increased the percentage of cells in S phase, and decreased the percentage of cells in G2/M phase, whereas the same concentration was ineffective in OCI-AML3 cells (Figure 2B). These results suggest that primary bone marrow cells are more sensitive than OCI-AML3 cells to the anti-proliferative and pro-apoptotic effects of FUS.

## 3. Discussion and Conclusions

Endophytic fungi exert numerous protective effects on plants and, through fungi–plant interactions, stimulate the production of important secondary metabolites. These substances can not only be utilized in the herbal world but can also be exploited to influence human cell/organ functions. This influence can be either positive or negative; although fungal metabolites can be used as drugs, they are also food contaminants that may exert toxic effects in the human body. 

As AFU and the methyl ether FUS, which are isolated from the endophytic fungi *Cladosporium* species reside in *Rauwolfia serpentina* plants, have anti-microbial and anti-tumor activities [12,21], we performed in-depth analysis of their putative anti-tumor or toxic effects and explored their mechanisms of action. Specifically, we compared the effects of FUS and AFU to that of DMSO vehicle to ascertain whether these compounds can be exploited therapeutically without exerting toxic effects on primary cells by evaluating inhibition of proliferation and escape from apoptosis, which can confer cancer resistance against many types of drugs.

Our results confirm the efficacy of FUS and AFU in blocking the growth of hematologic cancer cell lines by both promoting cell apoptosis and inhibiting cell cycle progression through the involvement of p21 and p53. These are two fundamental molecules in proliferation and apoptosis [22,23]. p21 inhibits Cdk-activating kinase (CAK) [24] in G2 phase. p21 also binds with the cyclin B1-Cdk1 complex as a consequence of genotoxicity, thus inhibiting activation by Cdc25 and CAK [25], and can determine G2 block by degradation of cyclin B1 in the presence of DNA damage [26,27]. The involvement of p21 was also putatively confirmed by the participation of MAPK molecules that regulate p21 expression and function. Phosphorylation by p38 increases p21 stability [28], whereas phosphorylation by ERK2 decreases p21 stability by promoting its degradation [29]. In addition, phosphorylation of Akt is responsible for the transfer of p21 from the nucleus to the cytoplasm in breast tumors [30]. The lack of a significant decrease in p21 expression as a consequence of p38 inhibition can be explained if we consider that the expression of p21 is the result of coordination between increased activation of p38 and decreased activation of both ERK and Akt. Thus, acting only on p38 would be insufficient to modify p21 expression. However, it is also possible that p38 activation is not involved in p21 expression in our experimental setting. 

Recent advances in understanding p21 function suggest that it can be either beneficial or harmful to cells. p21 is beneficial when it acts in concert with p53 as a consequence of DNA damage or stress. In these circumstances, p21 transiently blocks cell cycle progression leading to senescence or apoptosis, thus protecting the organism from damaged or mutated cells. However, when functional p53 is scarce, p21 promotes the escape of cells from senescence or apoptosis, thus creating conditions for the development of cancerous cells [16]. From this perspective, the mechanisms of actions of FUS and AFU are interesting, as AFU up-regulates p21 but not p53, suggesting a harmful mechanism. By contrast, FUS up-regulates both p21 and p53, but the latter only at higher concentrations. This suggests that the role of FUS depends on its dosage; higher doses are protective, whereas lower doses are harmful and similar to the effects of AFU. The protective effect of p21 could also be explained in terms of decreased activation of Akt, which can increase the nuclear location of p21 associated with its tumor suppressor activity [16]. 

We also dissected a second hallmark of cancer or toxicity: elusion of apoptosis. During the apoptotic process, the executioner caspase-3 is activated by at least two different pathways. The mitochondrial (i.e., intrinsic) pathway leads to sequential release of cytochrome c from mitochondria and activation of caspase-9, which directly cleaves and activates caspase-3. The second (i.e., extrinsic) pathway involves activation of caspase-8, which also directly cleaves and activates caspase-3 [20]. The absence of caspase-9 activation by the tested compounds is notable because it commonly participates in p21-induced apoptosis. This lack of caspase-9 activation and intrinsic pathway contribution was verified by a lack of changes in the expression of Bcl-xL or PUMA, which are proteins involved in the intrinsic apoptosis pathway. By contrast, FUS increased the activation of caspase-8 through elevated production of FasL. This is a novel finding, as FasL expression is usually not associated with p21 expression. We hypothesize that FUS can provide cells undergoing p21-dependent senescence with an escape mechanism from apoptosis through activation of the extrinsic pathway. However, additional experiments are needed to define the exact mechanisms induced by FUS, which could clarify which cell death pathway is activated by AFU, as caspase-3 was not activated by this compound. AFU could induce cell death via other pathways, such as necrosis or necroptosis, which are two mechanisms of caspase-independent cell death. Alternatively, AFU could stimulate a yet unknown mechanism of cell death.

Another relevant issue is the role of MAPK pathway in FUS-induced cytotoxicity. In our experimental setting, FUS-dependent apoptosis does not seem to be a direct consequence of the MAPK pathway because p38 inhibition does not decrease apoptosis, whereas activation of the ERK and Akt branches of the MAPK pathway, which could promote apoptosis [31,32], were significantly reduced by FUS.

The central query arising from this work is whether the growth arrest effects of FUS and AFU could be utilized for anti-cancer therapy or whether these compounds induce cell toxicity. To partially address this, we treated additional cell lines and primary mouse bone marrow cells with FUS and found diverse responses, suggesting that the effects of FUS are at least somewhat specific to OCI-AML3 cells. We do not know the cause of the variation between different cell lines in their response to FUS, which would need to be investigated in future studies. Although its effect on primary bone marrow cells raises doubt about the utilization of FUS for anti-leukemia therapy, it is important to consider that immortalized cell lines are even more resistant to therapy than primary tumor cells. Thus, in vivo experiments using animal models of cancer are needed to help gauge whether these compounds can be exploited for therapeutic purposes. 

In conclusion, we showed that FUS and AFU derived from the endophytic fungi *Cladosporium* species isolated from *Rauwolfia serpentina* exert anti-proliferative and pro-apoptotic effects on tumor and primary cells, in part due to MAPK-dependent up-regulation of p21. Further studies are needed to clarify whether these compounds could be used in cancer therapy.

## 4. Materials and Methods 

### 4.1. Isolation of Compounds

The methyl ethers FUS and AFU were isolated as previously reported [12]. A *Cladosporium* species, internal strain No. RSBE-3, which had been isolated following surface sterilization from the barks of the plant *Rauwolfia serpentina*, was cultivated at room temperature for 21 days on potato dextrose agar medium. The culture medium was extracted three times with ethyl acetate to obtain crude extract (3.0 g). The crude extract was subjected to column chromatography for fractionation on silica gel using gradients of petroleum ether/dichloromethane, dichloromethane, gradients of dichloromethane/methanol, and methanol to provide a total of 22 fractions. These fractions were screened by thin-layer chromatography (TLC) on silica gel under ultraviolet light in both short (254 nm) and long (365 nm) wavelengths and by spraying with vanillin–H_2_SO_4_ spray reagents. The column fraction of petroleum ether/75% dichloromethane was subjected to column chromatography for further fractionation. Crystallization from petroleum ether/dichloromethane (50%) produced fine needles of AFU (5.62 mg). The column fraction of dichloromethane/methanol (50%) was subjected to column chromatography for further fractionation. Crystallization from dichloromethane/methanol (1.5%) produced fine needles of FUS (9.46 mg). 

AFU appeared as dark violet spots on the TLC plate. It was soluble in dichloromethane and chloroform and sparingly soluble in methanol. R*_f_* 0.43 (toluene/5% EtOH); ^1^H NMR (500 MHz, CDCl_3_): δ 1.98 (3H, _S_, OCH_3_-7), 5.16 (2H, _S_, CH_2_-1), 5.92 (1H, _S_, H-4), 6.11 (1H, _S_, H-8), 12.57 (1H, _S_, OH-5), 12.97 (1H, _S_, OH-10), ^13^C NMR (125 MHz, CDCl_3_): δ 20.1 (C-11), 56.6 (C-12), 62.9 (C-1), 94.6 (C-4), 107.9 (C-9a), 109.9 (C-8), 110.9 (C-5a), 122.7 (C-10a), 132.9 (C-4a), 157.6 (C-10), 157.6 (C-5), 159.9 (C-7), 161.5 (C-3), 177.8 (C-6), 182.9 (C-9). ESIMS: *m/z* = 289 [M + H]^+^.

FUS appeared as dark quenching spots on the TLC plate. It was soluble in dichloromethane and chloroform and sparingly soluble in methanol. R*_f_* 0.44 (toluene/20% EtOAc); ^1^H NMR (500 MHz, CDCl_3_): δ 1.53 (3H, _S_, CH_3_-11), 2.65 (1H, dt, *J*_4,4_ = 18.0 Hz, *J*_4,1_ = 2.0 Hz, H-4), 2.99 (1H, dd, *J*_4,4_ = 18.0 Hz, *J*_4,1_ = 1.5 Hz, CH_3_-4), 3.30 (3H, _S_, OCH_3_-13), 4.54 (1H, dt, *J*_1,1_ = 17.8 Hz, *J*_1,4_ = 2.7 Hz, H-1), 4.85 (1H, dd, *J*_1,1_ = 17.8 Hz, *J*_1,4_ = 1.5 Hz, H-1), 6.15 (1H, _S_, H-8), 12.63 (1H, _S_, OH-5), 12.91 (1H, _S_, OH-10), ^13^C NMR (125 MHz, CDCl_3_): δ 22.8 (C-11), 33.0 (C-4), 48.9 (C-2), 56.7 (C-13), 58.7 (C-1), 96.8 (C-3), 107.5 (C-9a), 109.6 (C-5a), 109.7 (C-8), 132.9 (C-4a), 137.2 (C-10a), 157.2 (C-7), 160.7 (C-5), 160.7 (C-10), 178.2 (C-6), 184.7 (C-9). ESIMS: *m/z* = 321 [M + H]^+^.

The purity of the compounds was confirmed by TLC. The chromatographs were exposed under UV light at 254 nm and 366 nm, which produced unique single spots for both compounds. Chromatographs were also stained with vanillin-sulfuric acid followed by heating, but no extra spots were seen, further confirming compound purity. The NMR spectrum of the compounds was very clear without extra signals, providing additional confirmation their purity.

### 4.2. Cell Culture and Characterization

C57BL/6 female mice were purchased from Envigo (Calco, Lecco, Italy), and 8–12-week-old mice were used for experiments. Mice were housed in an isolated colony (22 °C, 55% humidity, 12-h/12-h light cycle) and provided with laboratory chow and acidified (pH 2.4) water ad libitum. All procedures involving mice were approved by the ethical committee (code number 462/2015 PR, 3 June 2015) of the University of Perugia. 

After removing muscle tissue from femurs and tibias, both ends of the bones were cut off, and the marrow was flushed out with a 25-gauge syringe filled with RPMI 1640 solution. The tissue was resuspended and passed through a mesh cell strainer (FALCON, Corning Incorporated, Corning, New York, NY, USA) to remove small pieces of bone and debris, and red blood cells were lysed with Gey’s solution (water solution with KHCO_3_ and NH_4_Cl) to recover ~50 × 10^6^ cells/mouse (one mouse/experiment was sacrificed). Bone marrow cells were counted using a hemocytometer. Single-cell suspensions (1 × 10^6^ cells/mL) were cultured in RPMI 1640 medium containing 10% fetal calf serum, 100 U/mL penicillin/streptomycin, 10 mM HEPES, 0.1% nonessential amino acids, and 1 mM sodium pyruvate (GIBCO Invitrogen, San Giuliano Milanese, Italy). Cells were maintained at 37 °C in 5% CO_2_ in flat-bottomed, 24-well plates (Thermo-Fischer Scientific, Waltham, MA, USA) for 24 h. In some experiments, p38 inhibitor (SB203580, Cell Signaling) at a concentration of 10 μM was added to FUS treatment for 24 h at the beginning of culture.

OCI-AML3, U937, HL-60, and Jurkat cells (all lymphoma or leukemia) were maintained in RPMI medium with 10% fetal bovine serum, 100 U/mL penicillin, and 100 μg/mL streptomycin at 37 °C in 5% CO_2_. All were purchased from ATCC, kept at logarithmic growth, and cultured in 24-well plates to assess their number and morphologies. Cultures, kept at 2 × 10^5^ cells/mL, were treated with different concentrations of DMSO or the test compounds at the final concentrations reported in the figures. Reported concentrations were chosen based on preliminary experiments. After 24 h, cell number was quantified using a hemocytometer.

### 4.3. Analysis of Cell Viability and Cell Cycle Progression

Cell viability and cell cycle sequence were examined by flow cytometry to measure the DNA amounts in nuclei colored with PI (Sigma-Aldrich, St. Louis, MO, USA). Briefly, cells were harvested by centrifugation and gently resuspended in 1.5 mL hypotonic PI solution (50 μg/mL in 0.1% sodium citrate plus 0.1% Triton X-100). Tubes were kept in the dark at 4 °C for 30 min. PI fluorescence of individual nuclei was measured by flow cytometry using a Coulter^®^ Epics XL-MCL™ Flow Cytometer (Beckman Coulter, Brea, CA, USA) and analyzed using FlowJo_V10 software (BD Biosciences, 2350 Qume Dr, San Jose, CA, USA).

### 4.4. Western Blotting and Analysis

Cells were pelleted in a conical tube by spinning at 1200 rpm for 5 min at room temperature, after which the media was decanted and the pellet was kept on ice. The pellet was washed one time with 5–10 mL ice-cold PBS and spun at 1200 rpm for 5 min, after which the PBS was decanted and the excess supernatant was aspirated. The pellet was then placed in 30 μL RIPA lysis buffer (5 M NaCl, 0.5 M EDTA, pH 8.0, 1 M Tris, pH 8.0, NP-40 (IGEPAL CA-630), 10% sodium deoxycholate, 10% SDS, dH2O) supplemented with protease (Sigma-Aldrich) and phosphatase (Thermo-Fisher Scientific) inhibitor cocktails. The lysate was incubated in ice for 30 min and centrifuged at 12,000 rpm for 5 min at 4 °C. The supernatant was collected into new microtubes, and protein concentration was determined by the bicinchoninic acid method. Proteins were separated by 12% or 15% sodium dodecyl sulfate-polyacrylamide gel electrophoresis (SDS-PAGE) and evaluated by Western blotting. Primary antibodies were polyclonal anti-caspase-3 (Cell Signaling, Danvers, MA, USA), anti-caspase-8 monoclonal antibody (mAb; clone 12F5, Enzo Life Sciences, Farmingdale, NY, USA), anti-caspase-9 mAb (clone ICE-LAP6, Mch6, Cell Signaling), anti-p21 mAb (clone 12D1, Cell Signaling), polyclonal anti-p44/42 MAPK (Erk1/2, Thr202/Tyr204, Cell Signaling), polyclonal anti-p44/42 MAPK (Erk1/2, Cell Signaling), polyclonal anti-phospho-p38 MAPK (Thr180/Tyr182, Cell Signaling), polyclonal anti-p38 MAPK (Cell Signaling), anti-p53 mAb (clone FL-393:sc-6243, Santa Cruz Biotechnology, Santa Cruz, CA), anti-Puma mAb (clone D30C10, Cell Signaling), polyclonal phosphor-Akt (Cell Signaling), polyclonal anti-Akt (Cell Signaling), anti-Bcl-xL mAb (clone 54H6, Cell Signaling), anti-GAPDH mAb (clone 2D9, OriGene, Rockville, MD, USA), and polyclonal anti-laminin B1 (Abcam, Cambridge, UK). Secondary antibodies were labeled with horseradish peroxidase (Pierce/Thermo-Fisher Scientific, Waltham, MA, USA). Antigen-antibody complexes were detected by enhanced chemiluminescence following the manufacturer’s instructions (Millipore, Billerica, MA, USA). Western blotting films were scanned, and band signal intensities were determined using ImageJ software (National Institutes of Health, Bethesda, MD, USA). p38 inhibitor was SB203580 (Cell Signaling) used at a concentration of 10 μM.

### 4.5. qRT-PCR

Generation of cDNA was performed in triplicate using a QuantiTect Reverse Transcription kit (Qiagen). All reactions were performed using an ABI-7300 Real-Time Cycler, and amplification was performed using TaqMan Assay (Hs00181225 for FasL, Hs00174128 for TNF, Hs00183225 for GITR-L, Hs00169409 for 4-1BBL, and eukaryotic 18S rRNA as an endogenous control).

### 4.6. Statistical Analysis

Statistical analysis was performed using GraphPad Prism 6. Differences between groups were evaluated using Mann–Whitney U tests. Differences were considered statistically significant as follows: * *p* < 0.05; ** *p* < 0.01; *** *p* < 0.001.
